# Simulation of Dense Star Map in Deep Space Based on Gaia Catalogue

**DOI:** 10.3390/s26061945

**Published:** 2026-03-19

**Authors:** Puzhen Li, Guangzhen Bao, Ziwei Zhou, Jinnan Gong

**Affiliations:** 1 Research Center for Space Optical Engineering, Harbin Institute of Technology, Harbin 150001, China; 25b321003@stu.hit.edu.cn (P.L.); guangzhenbao@stu.hit.edu.cn (G.B.); 2 Beijing Institute of Space Mechanics and Electricity, Beijing 100094, China

**Keywords:** Earth-Moon Lagrangian points, optical simulation, star field simulation, survey telescope

## Abstract

High-fidelity star field simulation is paramount for target detection and space situational awareness (SSA) in geostationary and deep-space environments. However, accurately modeling the synergistic effects of ultra-dense stellar backgrounds and complex platform perturbations remains a formidable challenge. This paper proposes an integrated simulation framework that leverages the Gaia catalog to generate high-precision stellar environments. The core methodological novelty lies in the end-to-end coupling of a full optoelectronic imaging chain with dynamic platform disturbances, effectively bridging the gap between theoretical orbital dynamics and realistic sensor responses. Distinguishing itself from conventional models, our approach uniquely integrates radiative transfer and high-fidelity noise suites—including photon shot noise and non-uniform stray light—while utilizing the Gaia catalog to achieve unprecedented precision in simulating dim stars at low magnitudes. The fidelity of the proposed model was quantitatively validated against empirical data from a ground-based wide-field telescope (GTC). Experimental results, derived from multiple simulation realizations, demonstrate high consistency with real-world observations, achieving a Signal-to-Noise Ratio (SNR) error of less than 10% and a sub-pixel centroiding accuracy exceeding 0.01 pixels. This work provides a robust, high-fidelity data synthesis tool that significantly advances the development of target detection algorithms and the performance optimization of space-based optical sensors.

## 1. Introduction

With the rapid development of aerospace technology, the scope of human space activities has gradually expanded from Low Earth Orbit (LEO) toward more distant outer space. This expansion brings increasingly complex security challenges. On the one hand, the proliferation of defunct satellites, rocket bodies, and fragmentation debris in high-altitude orbits poses a severe threat to operational spacecraft. Due to their long distances and faint signatures, traditional ground-based monitoring methods struggle to achieve all-time, high-precision cataloging and early warning capabilities. On the other hand, the risk of impact from deep-space threats, namely Near-Earth Objects (NEOs), is ever-present. Consequently, establishing a forward-looking deep-space defense system has become a focus for nations worldwide.

Against this background, enhancing High-Orbit Space Situational Awareness (SSA) capabilities is particularly critical. Compared to ground-based systems, which are affected by atmospheric turbulence and limited observation windows, space-based optical surveillance platforms offer inherent advantages, such as immunity to cloud cover, all-weather operation, and close-proximity observation.

However, as the detection performance of space-based optical systems improves, the influence of the stellar background also gradually increases. In the early optical era, the detection limit for stars was primarily determined by the light-gathering capability of the human eye and small telescopes; Ptolemy’s star catalog from 150 AD only recorded about 1000 stars, with a limiting magnitude of approximately 6. With the application of photographic technology, the stellar detection limit rapidly increased, and late 20th-century catalogs like the Digitized Sky Survey (DSS) [1] and Guide Star Catalog (GSC) [2] could reach a detection limit of magnitude 20. Today, with the widespread use of devices like CCD and CMOS, the Sloan Digital Sky Survey (SDSS) [3] can achieve a limiting magnitude of 22.5 in the r-band. Therefore, stronger detection capability inherently implies more stellar interference.

Existing star field simulations typically rely on catalogs like Tycho-2 or UCAC4, which are sufficient for basic attitude determination but inadequate for high-fidelity situational awareness. Moreover, conventional frameworks often employ simplified point spread functions (PSFs) and decoupled kinematic models, causing poor energy concentration and significant Signal-to-Noise Ratio (SNR) errors when rendering dense, faint star fields. Accurately detecting dim high-orbit targets demands physically rigorous simulations of these faint backgrounds.

To address this, we propose an advanced simulation framework built strictly upon a complete optoelectronic imaging chain. By utilizing the Gaia catalog, our method accurately renders low-magnitude stars, significantly enriching background complexity. Unlike traditional approaches, we tightly couple precise energy transfer and dynamic motion models directly into the imaging link. This integration yields superior physical fidelity—demonstrated by higher energy concentration and substantially lower SNR errors—providing a robust data foundation for evaluating dim-target detection algorithms in deep space.

This paper directly addresses this issue by proposing a star catalog-based simulation technique for dense stellar backgrounds.

The subsequent structure and main content of this paper are organized as follows:

1. Chapter 2 introduces and analyzes the relevant theoretical concepts involved in the simulation technique.
2. Chapter 3 details the modular construction of the actual simulation model.
3. Chapter 4 presents the experimental design of the simulation model, along with a fidelity assessment using real observational data from the Ground-based Wide-field Telescope (GTC). This chapter also provides simulation schemes and results for several specific scenarios.

## 2. Theoretical Analysis

This chapter establishes a comprehensive theoretical framework for a full-link physical simulation model tailored for space-based observation systems. This model serves as the foundation for subsequent implementation and performance characterization. To meet the rigorous detection requirements for high-orbit space debris and deep-space small celestial bodies, the analysis encompasses the entire physical chain—extending from orbital dynamics resolution to optoelectronic imaging conversion.

### 2.1. Analysis of Target Characteristics

**Figure 1:**
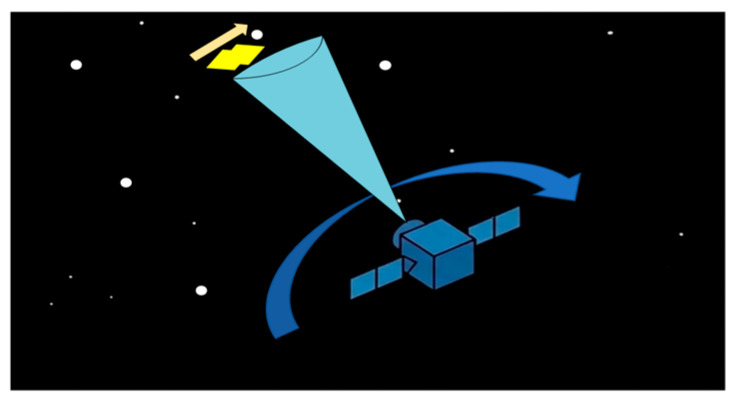
Schematic of Relative Motion between Observation Platform, Space Target, and Stellar Background.

A systematic characterization of the targets, stellar backgrounds, and imaging mechanisms is provided. As illustrated in Figure 1, the simulation explicitly accounts for the relative motion between the observation platform and space target. The arrows in the figure indicate the direction of this motion. Due to the immense distances involved, the stellar background is treated as a stationary celestial reference frame, while the dynamic interaction is primarily dictated by the satellite’s orbital maneuvers and attitude perturbations.

#### 2.1.1. Target Motion State

To support the high-fidelity simulation, the relative motion between the observation platform and the space target is first modeled. In an ideal scenario, the interaction between the Earth and the space target is simplified as a two-body problem, where the target is assumed to move in a circular orbit. Under these assumptions, the orbital velocity v and the angular velocity w of the target are defined as [4]:

(1) $$\begin{array}{l} v=\sqrt{\frac{\mu }{R_{earth}+H}}\\ w=\sqrt{\frac{\mu }{(R_{earth}+H{)}^{3}}} \end{array}$$

where μ is the Geocentric Gravitational Constant, $R_{earth}$ denotes the mean radius of the Earth, and H represents the orbital altitude of the target. The relative distance ρ between the observation platform and the target can be derived using the Law of Cosines:

(2) $$\rho =\sqrt{{R_{obs}}^{2}+{\left(R_{earth}+H\right)}^{2}-2\cdot R_{obs}\cdot (R_{earth}+H)\cdot \cos\theta }$$

where $R_{obs}$ is the distance from the Earth’s center to the observation platform (e.g., the Earth-Moon distance for specific lunar-based scenarios), and θ is the angular separation between the platform and the target relative to the geocenter.

In the triangle defined by the observation platform, the space target, and the Earth, the Law of Sines is applied as follows:

(3) $$\theta_{1}=\arcsin[\frac{\rho }{\sin(\theta )}\cdot (r_{earth}+H)]$$

$\theta_{1}$ is the angle subtended by the Earth and the space target at the observation platform. Therefore, the angle $\theta_{2}$, which corresponds to the tangential component of the space target relative to the observation platform, is expressed as:

(4) $$\theta_{2}=90-[(90-\theta )-\theta_{1}]$$

Consequently, the angular velocity of the space target relative to the observation platform can be expressed as:

(5) $$\begin{array}{l} w_{O-T}=\frac{v\cdot \cos\theta_{2}}{\rho }\\ =\frac{\sqrt{\frac{u}{r_{earth}+H}}\cdot \cos(90-[(90-\theta )-\arcsin[\frac{\rho }{\sin(\theta )}\cdot (r_{earth}+H)]])}{\sqrt{{R_{earth-moon}}^{2}+{\left(r_{earth}+H\right)}^{2}-2\cdot R_{earth-moon}\cdot (r_{earth}+H)\cdot \cos\theta }} \end{array}$$

In the actual simulation process, we employ two modes to simulate the target’s motion trajectory: directly inserting virtual trajectories onto the image plane, and calculating the target trajectory based on TLE data and then mapping it onto the image plane. The former method involves directly generating several sets of trajectories for the target moving on the image plane (with controllable trajectory types). The latter requires calculation combining the camera’s internal and external parameters.

#### 2.1.2. Target Energy Distribution Analysis

Due to the target’s significant distance from the detector, it forms a point source on the image plane, exhibiting an imaging distribution essentially identical to that of a star. The energy distribution of a single stellar image is described by the response function of a point source, which is the Point Spread Function (PSF). The Optical Transfer Function (OTF) is its corresponding Fourier Transform. The stellar light intensity distribution is simulated using the circular aperture diffraction model, yielding the following equation:

(6) $$I(\Phi {)=I}_{0}{\left[\frac{2J_{1}(\Phi )}{\Phi }\right]}^{2}$$

where $I_{0}$ is the peak intensity, and $J_{1}$ is the first-order Bessel function

**Figure 2:**
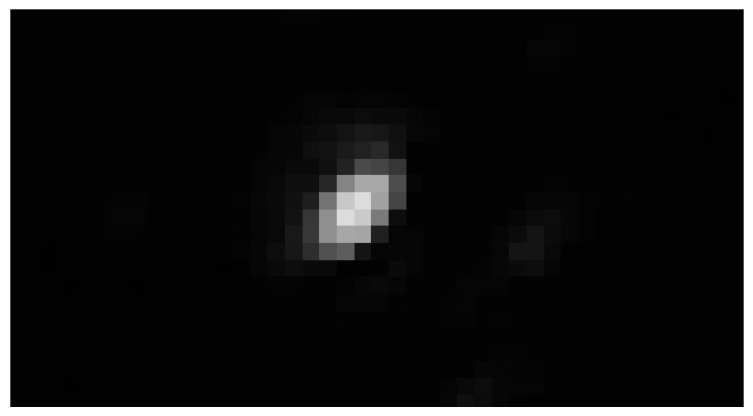
Diagram of Streaked Target.

In ideal star maps, stellar targets and faint small targets are represented as point sources. When a survey camera utilizes long integration times—typically when staring at stars to observe faint objects—targets with relative motion develop motion smear (or streaking). The motion (smear) of the streaked target, measured in pixels per frame interval, is calculated as:

(7) $$dN=\frac{wt}{\varepsilon }$$

where w is the angular velocity of the target relative to the image plane; and ε is the angular resolution of a single detector pixel. The simulation results for the streaked target are shown in Figure 2.

### 2.2. Analysis of Stellar Intensity

#### 2.2.1. Stellar Intensity Analysis

Stars are approximated as point sources located at an infinite distance from the detector. In the dark background of deep space, their radiation propagates through the imaging chain, influenced by the optical system’s Optical Transfer Function (OTF) and the resulting Point Spread Function (PSF). Consequently, the energy is finally dispersed into a Gaussian spot (or blob) on the image plane. However, the actual size of the resulting image may be affected by multiple factors, including the optical system characteristics, the detector, and the jitter (vibration) of the target and the observation platform. Its two-dimensional Gaussian distribution on the image is expressed as [5]:

(8) $$f(x,y)=\frac{1}{2\pi \sigma_{x}\sigma_{y}}\exp\left(-\frac{{\left(x-\mu_{x}\right)}^{2}}{2\sigma_{x}^{2}}-\frac{{\left(y-\mu_{y}\right)}^{2}}{2\sigma_{y}^{2}}\right)$$

where $\mu_{x}$ and $\mu_{y}$ are the distribution means (i.e., the pixel center); and $\sigma_{x}$ and $\sigma_{y}$ are the distribution standard deviations (i.e., the standard deviation of the PSF).

#### 2.2.2. Band Impact Analysis

The simulation magnitude is based on the G-band magnitude data provided in the Gaia catalog. This band is the core photometric system of the Gaia catalog, covering 330~1050 nm, with the effective response mainly concentrated between 400~900 nm, and peak sensitivity at approximately 673 nm. The specific value of the magnitude is calculated by integrating the star’s photon flux in the G-band [6], using the formula:

(9) $$G=-2.5\log_{10}(F_{g})+Z_{g}$$

$F_{g}$: The measured flux in the G-band (instrument-calibrated)
$Z_{g}$: The zero-point of the G-band, calibrated using standard stars

To simulate the star’s appearance on the detector, the catalog magnitude G is converted into the corresponding focal plane flux (pixel intensity) using the following relationship:

(10) $$I=\Phi_{0}\cdot 10^{-0.4G}\cdot A\cdot \tau \cdot \eta \cdot ∆t$$

where $\Phi_{0}$ is the reference flux for a zero-magnitude star in the G-band, A is the effective aperture area, τ is the optical transmittance, η is the quantum efficiency, and ∆t is the integration time. This ensures a physically consistent mapping from catalog data to simulated digital numbers (DN).

Within the defined spectral range, the diffraction limit is evaluated to justify the PSF modeling. The maximum radius of the star’s Airy disk is calculated at the upper-bound wavelength of 900 nm as follows:

(11) $$r_{\max}=1.22\frac{\lambda_{\max}f}{D}\approx 2.32\mu m$$

where f is the focal length and D is the aperture diameter. In this simulation, these diffraction parameters are utilized to determine the kernel size of the Gaussian PSF, ensuring that the energy concentration of the simulated stars accurately reflects the theoretical resolution of the optical system.

Among the wavelength-related factors, the diffraction radius remains smaller than the pixel size even in the maximum diffraction state, satisfying the envelope requirement that the energy concentration is greater than 90%. Therefore, a central wavelength of 673 nm will be adopted for the subsequent simulation.

### 2.3. Imaging Model Analysis of Deep Space Exploration System

#### 2.3.1. Analysis of Factors Affecting Geometric Performance

The satellite orbital dynamics model is utilized to generate high-precision attitude and orbital parameters, which are essential for accurate platform positioning. To ensure simulation fidelity, the model accounts for various orbital perturbations and the precise timing offsets between different astronomical time systems (e.g., UTC, TAI, and TDT). Furthermore, system compensations are integrated to account for the sensor’s relative imaging velocity and viewing angle variations.

**Figure 3:**
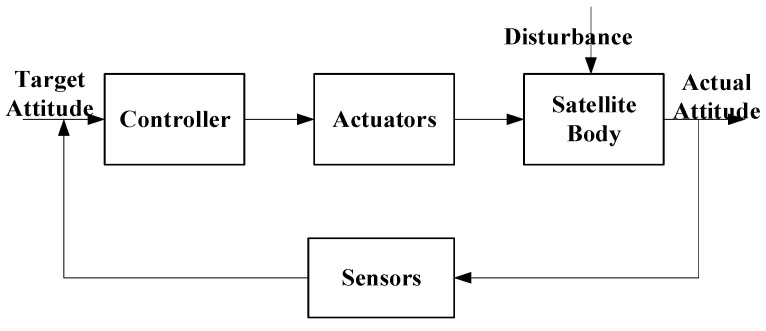
Control flow of satellite attitude.

The modeling of satellite attitude characteristics is tightly coupled with the closed-loop attitude control system (ACS), as illustrated in Figure 3. Rather than being treated as static parameters, the satellite attitude is dynamically simulated within the control loop. Importantly, the attitude control errors and high-frequency disturbances (e.g., micro-vibrations) generated by this model are directly propagated into the imaging chain. These disturbances are translated into time-varying line-of-sight (LOS) jitter and integrated over the exposure time, thereby simulating realistic motion blur and geometric distortion in the synthetic star maps. This coupling ensures that the simulated images accurately reflect the dynamic instability of the observation platform.

#### 2.3.2. Sampling Model Analysis

Discrete sampling is an inherent characteristic of all optoelectronic imaging systems [7]. The discrete distribution of the detector elements dictates that the scene is sampled in two spatial directions. The sampling model is given by:

(12) $$E\left(m,n\right)=\sum_{m=1}^{M}\sum_{n=1}^{N}\delta \left(x-mp_{x},y-np_{y}\right)\cdot S\left(x,y\right)$$

where $p_{x}$ is the spacing between adjacent detector elements in the x direction; and $p_{y}$ is the spacing between adjacent detector elements in the y direction.

#### 2.3.3. Analysis of Noise Models

Noise sources include: dark current noise (detector), shot noise (detector, circuit), readout noise (detector), amplifier noise (circuit), quantization noise (circuit), and pattern noise (detector) [8]. The first five are additive noise, and the last is multiplicative noise. The models for additive and multiplicative noise are as follows:

(13) $$S\left(m,n\right)=Se\left(m,n\right)+N\left(m,n\right)$$

(14) $$S\left(m,n\right)=Se\left(m,n\right)\left(1+r\left(m,n\right)\right)$$

Dark current typically originates from surface defects at the SiO_2_/Si interface, surface generation, thermal generation, and defects in the semiconductor manufacturing process. The dark current generated in a single pixel during the integration time, expressed in electrons, is:

(15) $$n_{dc.e^{-}}=t_{\mathrm{int}\;}\cdot J_{D}A_{d}/q$$

where $t_{\mathrm{int}\;}$ is the detector integration time; $J_{D}$ is the dark current density; $A_{d}$ is the detector pixel area; and q is the charge carried by one electron.

In addition to dark current density, the pixel area and integration time also influence the magnitude of dark current noise. Since the detector randomly generates electrons, the dark signal produces additional noise known as dark current shot noise, which is therefore described by the Poisson process:

(16) $$n_{dc.shot.e^{-}\;}=P(\Lambda )$$

where $\Lambda =n_{dc.e^{-}}$, shot noise is time-related, making it temporal noise.

Dark current and shot noise are generated in the integration process of the detector, and the magnitude is related to the integration time and the size of the detector.

Readout noise is generated during the charge transfer process, is independent of the signal, and is analyzed using a white noise model. Its value is usually provided in the detector manual (readout noise, noise equivalent electrons, or noise floor).

Amplifier noise includes on-chip amplifier noise and off-chip amplifier noise. On-chip amplifier noise mainly includes 1/f noise and white noise; off-chip amplifier noise is similar to on-chip amplifier noise and is treated as white noise here.

Quantization noise is generated during AD conversion. Its noise standard deviation is inversely proportional to the number of quantization bits; the more quantization bits, the lower the noise standard deviation, as shown in the formula below. This type of noise is treated as white noise.

(17) $$\sigma_{\mathrm{ADC}}^{2}=\frac{S_{\mathrm{saturation}}}{2^{n}\cdot 12}$$

where $S_{\mathrm{saturation}}$ is the saturation electron count, and n is the number of quantization bits.

Pattern noise primarily includes Fixed Pattern Noise (FPN) and Photo Response Non-Uniformity (PRNU).

Using $U_{DCNU}$ to represent dark current non-uniformity, the fixed pattern noise is:

(18) $$\sigma_{\mathrm{FPN}}=U_{DCNU}\times \sigma_{\mathrm{dark}}$$

Using $U_{PRNU}$ to represent photo response non-uniformity, the PRNU noise is:

(19) $$\sigma_{PRNU}=U_{PRNU}\times S_{e}$$

The two parameters, FPN and PRNU, typically refer to dark current non-uniformity and photo response non-uniformity, respectively [9]. The standard deviation used for calculation is usually obtained by dividing by 5~6 times.

The Poisson probability distribution model is:

(20) $$P\left(x\right)={\frac{\lambda^{x}e}{x!}}^{-\lambda }$$

The Gaussian probability distribution model is:

(21) $$P\left(x\right)=\frac{1}{\sqrt{2\pi }\sigma }e^{-{\left(x-\mu \right)}^{2}/2\sigma^{2}}$$

#### 2.3.4. Quantitative Model Analysis

The Digital Number (DN) value of the quantized digital image is given by:

(22) $$DN=\mathrm{int}\left(\frac{S}{LSB}\right)$$

where S is the electrical signal generated by the detector; LSB is the Least Significant Bit or quantization interval; and int is the integer truncation operator (or rounding-down operator). For uniform quantization, the LSB is shown below:

(23) $$LSB=\frac{S_{sa}}{2^{n}}$$

where $S_{sa}$ is the saturation electron count (or full-well capacity).

The aforementioned noise sources are integrated into the simulation following the physical image-formation sequence. First, photon shot noise is modeled as a Poisson process during signal accumulation. Next, dark current and FPN are added to simulate thermal and non-uniformity effects. Finally, readout noise and quantization errors are introduced during the signal conversion and digitization stages. This step-by-step approach ensures the synthetic images accurately reflect the stochastic and systematic uncertainties of the detector.

## 3. The Establishment of Simulation Model

The complete image simulation process begins by inputting the orbital data of the observation satellite and targets, alongside star catalog data. Subsequently, the orbital position data for the satellite, targets, and stars are resolved. Within the deep-space background simulation module, simulation is performed based on the characteristics of the targets and the stellar background to obtain object-space simulation data. As the optical telescope in the detection system collects optical signals from space targets, non-target optical signals from the space environment (such as solar radiation) are captured simultaneously in the form of stray light; therefore, it is necessary to model the stray light entering the system [10]. Finally, the detector noise generated during the optoelectronic signal conversion process is modeled to complete the imaging link modeling of the deep-space detection system.

**Figure 4:**
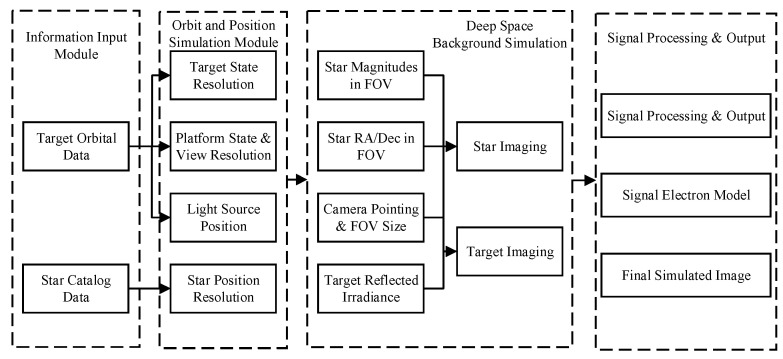
Flow chart of the overall plan.

Regarding the simulation content, the settings are divided into objective and subjective factors. Objective factors refer to results that are not influenced by parameter inputs, primarily involving the information input and orbital position simulation modules, including platform coordinates, star catalogs, and target orbital data. Subjective factors are simulation components influenced by subjective inputs, mainly including the deep-space background simulation module, stray light modeling, and the signal electron count model, which incorporate camera parameters, noise parameters, and other variables, as shown in Figure 4.

To ensure the authenticity of the relative positions between the stars, the observation platform, and the observation targets, the inputs for the simulation model consist of public TLE data and star catalogs. For the simulation of stellar positions and energy within dense star field images, a more extensive star catalog is utilized; specifically, the Gaia catalog is adopted as the primary input source for stellar position and magnitude information.

### 3.1. Orbit Position Simulation Module

#### 3.1.1. Resolution of Star Positions on the Image Plane

Stars can be considered to be at an infinite distance from both the detector and the Earth’s center. Thus, the phase angle of a star relative to the satellite in the Inertial Coordinate System is equivalent to the star’s Right Ascension (Ra) and Declination (Dec) data [11].

(24) $$\left\{\begin{array}{l} x_{im}^{I}=\cos\mathrm{Ra}\\ y_{im}^{I}=\sin\mathrm{Ra}\\ z_{im}^{I}=\sin\mathrm{Dec} \end{array}\right.$$

where RA is the Right Ascension in star catalog coordinates, Dec is the Declination in star catalog coordinates, and $x_{im}^{I},y_{im}^{I},z_{im}^{I}$ is the corresponding unit direction vector of the star relative to the detector in the Earth-Centered Inertial (ECI) frame.

After inputting Ra, Dec, and magnitude data from the Gaia catalog, the first step is to resolve the star’s position on the image plane. The calculation steps are as follows [12]:

1. RA and Declination (Dec) are the fundamental parameters of the celestial coordinate system used to describe the positions of celestial bodies on the celestial sphere.

**Figure 5:**
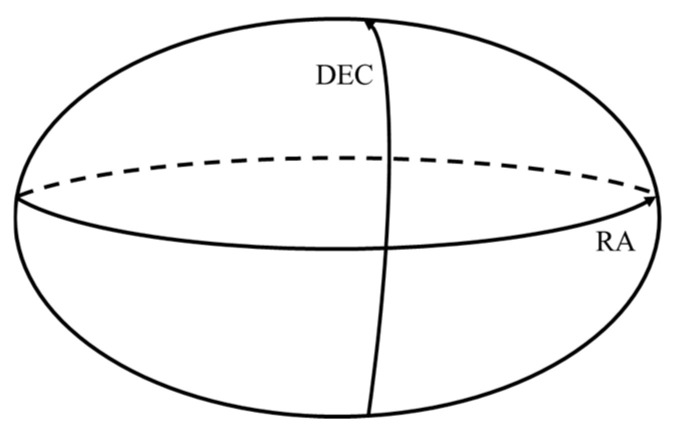
Schematic of Right Ascension (RA) and Declination (Dec).

Right Ascension (RA) is analogous to longitude on Earth; it uses the vernal equinox as the zero point and is measured eastward along the celestial equator. It is typically expressed in time units (hours) or degrees, ranging from 0° to 360°, as shown in Figure 5.

Declination (Dec) is analogous to latitude, representing the angular distance of a celestial body relative to the celestial equator, ranging from −90° to +90°.

The celestial coordinate system is a system used to describe the positions of objects on the celestial sphere. It is centered on the Earth, projecting the positions of celestial bodies onto an imaginary sphere. This system is closely related to the Earth’s axis of rotation and the equatorial plane. Conversion from RA and Dec to Celestial Rectangular Coordinates [13]:

(25) x=cos(δ)⋅cos(α)

(26) y=cos(δ)⋅sin(α)

(27) z=sin(δ)

where α, δ: are the input Right Ascension and Declination in degrees. ${\left[x,y,z\right]}^{T}$ represents the output unit direction vector in the celestial coordinate system.

2. Transformation from Celestial Coordinates to the Satellite Inertial Coordinate System

As the satellite moves in space, its inertial reference frame is typically aligned with the celestial coordinate system (the J2000 coordinate system is adopted in this paper). In this step, the celestial rectangular coordinate system obtained in the first step can be directly utilized. Specifically, the unit vector of the star’s coordinates in the inertial reference frame is defined as: ${\left[x,y,z\right]}^{T}$

3. Transformation from the Inertial Coordinate System to the Satellite Body Coordinate System

The satellite body coordinate system is typically defined such that the optical axis is aligned with the *Z*-axis, while the *X* and *Y* axes lie within the image plane. The satellite’s attitude is defined by a rotation matrix and a displacement vector. The formula for mapping coordinates from the J2000 coordinate system to the satellite body coordinate system is as follows:

(28) $$P_{\mathrm{satellite}}=R_{\mathrm{sat}}\cdot P_{\mathrm{inertial}}+T_{\mathrm{sat}}$$

where $R_{\mathrm{sat}}$ is a 3 × 3 rotation matrix, and $T_{\mathrm{sat}}$ is a 3 × 1 translation vector describing the displacement from the origin of the reference coordinate system to the origin of the satellite coordinate system. This step yields the final output: the unit direction vector of the star expressed in the satellite body coordinate system ${\left[x\prime ,y\prime ,z\prime \right]}^{T}$.

4. Perspective Projection from the Satellite Coordinate System to the Image Plane Coordinate System

A perspective projection is employed to transform the three-dimensional satellite body coordinates into two-dimensional image plane coordinates, specifically onto the normalized unit plane:

(29) $$x^{\prime \prime }=\frac{x^{\prime }}{z^{\prime }}$$

(30) $$y^{\prime \prime }=\frac{y^{\prime }}{z^{\prime }}$$

where $\left[x^{\prime \prime },y^{\prime \prime }\right]$ are the unit plane coordinates, representing the normalized image plane coordinates.

5. Transformation from Normalized Image Plane Coordinates to Pixel Coordinates

The normalized image plane coordinates are mapped to the actual pixel coordinates using the intrinsic parameters (or internal parameters) provided by the camera manufacturer:

(31) $$u=f_{x}\cdot x^{\prime \prime }+c_{x}$$

(32) $$v=f_{y}\cdot y^{\prime \prime }+c_{y}$$

where $f_{x}$ and $f_{y}$ denote the ratio of the focal length to the pixel size in the x and y directions, respectively. $c_{x}$ and $c_{y}$ represent the pixel coordinates of the principal point (the point where the optical axis intersects the image plane). The final output is the pixel coordinates (u,v).

#### 3.1.2. Target Image Plane Position Resolution

The resolution of the target position on the image plane requires mapping the target coordinates in the Earth-Centered Inertial (ECI) frame, derived from TLE data, onto the image plane coordinates [14].

1. Transformation from ECI Coordinate System to Orbital Coordinate System

In the ECI frame, let the target position vector be r and the satellite position vector be S. The target position vector transformed from ECI to ORB is given by:

(33) $$\;r_{o}=BA(r-S)$$

where the coordinate rotation matrix is:

(34) $$A=R_{z}(u)R_{x}(i)R_{z}(\Omega )$$

In this expression, i denotes the orbital inclination, Ω represents the Right Ascension of the Ascending Node (RAAN), and $u=\omega +\theta_{1}$ is the argument of latitude.

The axis swap matrix is defined as:

(35) $$B=\left[\begin{array}{ccc} 0 & 1 & 0\\ 0 & 0 & -1\\ -1 & 0 & 0 \end{array}\right]$$

2. Transformation from Orbital Coordinate System to Satellite Body Coordinate System

The satellite body coordinate system is obtained by rotating the orbital coordinate system through three directions: roll, pitch, and yaw. Let the three Euler angles be ψ, θ, φ, and the rotation matrix be $T_{ORB}^{BODY}$. Then:

(36) $$T_{ORB}^{BODY}=R_{y}(\theta )R_{x}(\psi )R_{z}(\varphi )$$

If the coordinates of the target in the orbital and body frames are $r_{O}$ and $r_{B}$ respectively, then:

(37) $$r_{B}=T_{ORB}^{BODY}r_{O}$$

3. Transformation from Satellite Body Coordinate System to Sensor Coordinate System

The satellite body coordinate system aligns with the sensor coordinate system after the following rotations: A counter-clockwise rotation of angle $\alpha_{p}$ around the *Z*-axis; A counter-clockwise rotation of angle $e_{p}$ around the *Y*-axis. The corresponding rotation matrix is:

(38) $$T_{BODY}^{SEN}=R_{y}(e_{pt})R_{z}(\alpha_{pt})$$

If the target coordinates in the body and sensor frames are $r_{B}$ and $r_{S}$, then:

(39) $$r_{S}=T_{BODY}^{SEN}r_{B}$$

4. Transformation from Sensor Coordinate System to Image Plane Coordinate System

The position in the sensor coordinate system undergoes projection and discretization to yield the image plane position. Let the target coordinates be $r_{s}={\left[x_{s},y_{s},z_{s}\right]}^{T}$. The projected position is:

(40) $$r_{P^{\prime }}={\left[\begin{array}{ll} \frac{x_{S}}{z_{S}} & \frac{y_{S}}{z_{S}} \end{array}\right]}^{T}$$

Discretization is performed using the following method:

(41) $$r_{P}={\left[\left\lfloor \frac{1}{p}\arctan\left(\frac{x_{s}}{z_{s}}\right)-\frac{1}{2}\right\rfloor \left\lfloor \frac{1}{p}\arctan\left(\frac{y_{S}}{z_{S}}\right)-\frac{1}{2}\right\rfloor \right]}^{T}$$

where p is the camera resolution, and $\left\lfloor \cdot \right\rfloor$ denotes the floor operation (taking the lower limit).

**Figure 6:**
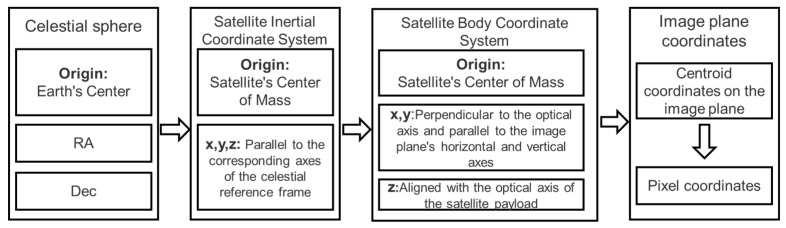
Schematic of Coordinate System Definitions and Transformation Processes.

The complete calculation pipeline for stellar image plane coordinates and the corresponding coordinate system definitions are illustrated in Figure 6.

#### 3.1.3. High-Precision Centroid Simulation Based on Bicubic Interpolation

Compared with bilinear interpolation, which considers only a 2 × 2 neighborhood and lacks derivative continuity, bicubic interpolation employs a 4 × 4 neighborhood integrated with cubic polynomials. By fitting the intensity variation trend among 16 adjacent pixels, this approach ensures the continuity of both the reconstructed surface and its first-order derivatives. Consequently, it effectively preserves high-frequency stellar profiles and minimizes sub-pixel centroiding errors. For high-fidelity simulations leveraging the Gaia catalog, such an approach is essential to maintaining a centroid accuracy within 0.01 pixels [15]. For a target point (x,y), the interpolated pixel value f(x,y) is calculated as follows:

(42) $$f(x,y)=\sum_{i=-1}^{2}\sum_{j=-1}^{2}f(x_{i},y_{j}\;)\cdot W(x-x_{i}\;)\cdot W(y-y_{j}\;)$$

where $f(x_{i},y_{j}\;)$ represents the grayscale values of the 16 neighboring pixels, and *W* is the bicubic weighting function:

(43) $$W(t)=\left\{\begin{array}{ll} (a+2){\left|t\right|}^{3}-(a-3){\left|t\right|}^{2}+1 & if\left|t\right|\leq 1\\ a{\left|t\right|}^{3}-5a{\left|t\right|}^{2}+8a\left|t\right|-4a & if1<\left|t\right|<2\\ 0 & otherwise \end{array}\right.$$

In this formula, a is an adjustment parameter taken as −0.5 during the simulation, and t is the horizontal or vertical distance between the target point and the neighboring pixel. Through bicubic interpolation, the centroid simulation error can be controlled within 0.01 pixels.

For a real image signal f(x), performing a Taylor expansion at x=0 yields:

(44) $$f(x)=f(0)+f^{\prime }(0)x+\frac{f^{\prime \prime }(0)}{2}x^{2}+\frac{f^{\prime \prime \prime }(0)}{6}x^{3}+O(x^{4})$$

The approximation error of bicubic interpolation originates from [16]:

(45) $$E(x)=\left|f(x)-\sum_{i=-1}^{2}f(i)W(x-i)\right|$$

when the third-order derivative of f(x) is non-zero, the error is primarily determined by the fourth-order term $O(h^{3})$:

(46) $$E(x)\approx \frac{f^{\prime \prime \prime }(0)}{6}\left|x^{3}-\sum_{i=-1}^{2}f(i)W(x-i)\right|$$

For △x∈[0,1), the maximum error is approximately $0.01\cdot f^{\prime \prime \prime }(0)$. For sub-pixel displacements (i.e., 0.01 pixels), the error term $E(△x)\propto {\left(0.01\right)}^{3}=10^{-6}$.

### 3.2. Energy Simulation Module

#### 3.2.1. Stellar Energy Simulation

When simulating the starry sky in a computer, the simulated star map is displayed in the form of a two-dimensional digital image. In addition to obtaining the position of the star point on the detector image plane, it is necessary to determine the grayscale value corresponding to the pixels. This involves converting the apparent magnitude of the observed star in the star catalog into the grayscale value on the detector image plane.

Stellar magnitudes are categorized into apparent magnitude and absolute magnitude. Apparent magnitude refers to the brightness of a celestial body as seen by an observer, while absolute magnitude is the brightness as seen from a distance of 32.616 light-years. The simulation data source utilizes the apparent magnitude. Based on the relationship where the irradiance decreases by 2.51 times for every one-level increase in magnitude [17], the expression relating magnitude to irradiance is:

(47) $$m_{i}=-2.51\mathrm{lg}\frac{E_{i}}{E_{0}}$$

That is:

(48) $$E_{i}=E_{0}\cdot 10^{-\frac{m_{i}}{2.51}}$$

where $m_{i}$ is the magnitude of the i-th observed star, $E_{i}$ is the irradiance of the i-th observed star, and $E_{0}$ is the irradiance of a zeroth-magnitude star. Using a linear calculation method, the relationship between star point grayscale and irradiance is:

(49) $$g_{i}=(E_{i}-E_{\min})\cdot \frac{2^{14}-g_{\min}}{E_{\max}-E_{\min}}+g_{\min}$$

where $g_{i}$ is the grayscale value of the i-th star, $E_{\max}$ is the maximum irradiance, $E_{i}$ is the magnitude of the i-th star, and $g_{\min}$ is the grayscale value corresponding to the minimum irradiance.

Stars are at vast distances from Earth and can be regarded as point sources for the observer. Due to inherent aberrations in the optical system, the grayscale of the star point diffuses, and the energy distribution of the image spot follows a Point Spread Function (PSF) model. By employing defocusing techniques to spread the star image over multiple pixels, the positioning accuracy of star extraction algorithms can reach sub-pixel levels (e.g., 1/10th of a pixel). To accurately simulate the star map, the grayscale dispersion caused by aberrations, defocusing, and glare must be considered. The energy distribution of the star image point is approximated by a two-dimensional Gaussian function model:

(50) $$g(x_{i},y_{i})=\frac{A}{2\pi \sigma^{2}}\cdot \exp[-\frac{{\left(x-x_{0}\right)}^{2}+{\left(y-y_{0}\right)}^{2}}{2\sigma^{2}}]$$

where $\left(x_{0},y_{0}\right)$ is the center of the Gaussian surface, $g(x_{i},y_{i})$ is the grayscale value at $\left(x_{i},y_{i}\right)$, A is the total energy (grayscale sum), and σ represents the radius of the diffusion spot.

#### 3.2.2. Target Energy Simulation

Target energy is determined via the Signal-to-Noise Ratio (SNR) and the noise background energy. The SNR of the imaging system is defined as the integrated SNR, which is the ratio of the total target energy to the mask energy. Thus, the total energy of the target image spot is:

(51) $$E_{tar}=SNR_{integrated}\times \iint E_{noise}(x,y)dxdy$$

Based on the analysis of the optical system’s Modulation Transfer Function (MTF)—assuming an off-axis three-mirror reflective configuration—the Point Spread Function (PSF) for target imaging can be derived as follows:

(52) $$PSF(x,y)={\left[\frac{2J_{1}(\frac{\pi r}{r_{0}})}{\frac{\pi r}{r_{0}}}\right]}^{2}$$

Consequently, the energy distribution of the target is expressed as:

(53) $$E(x,y)=E_{tar}{\left[\frac{2J_{1}(\frac{\pi r}{r_{0}})}{\frac{\pi r}{r_{0}}}\right]}^{2}$$

### 3.3. Establishment of MTF Models

The MTF chain encompasses various stages, including the optical system, detector, and satellite platform motion. The image degradation caused by satellite platform jitter cannot be modeled in isolation; it must be integrated with the detector’s temporal integration effect [18,19,20]. Treating the system as a linear system, the total system MTF is expressed as:

(54) $$MTF_{sys}=MTF_{opt}\cdot MTF_{cmos}\cdot MTF_{filter}\cdot MTF_{sat-cmos}$$

where $MTF_{opt}$, $MTF_{cmos}$, $MTF_{filter}$, and $MTF_{sat-cmos}$ represent the MTFs of the optical system, detector, circuitry, and the integrated satellite-detector platform, respectively.

#### 3.3.1. Optical System MTF

The optical MTF can be simulated using prior static transfer function data or by modeling effects such as diffraction, aberration, defocus, and fabrication/assembly errors:

1. Diffraction-limited MTF with Central Obscuration

For a circular aperture optical system with a central obscuration, the diffraction-limited MTF in the x-direction is given by

(55) $$MTF_{diff}=\frac{A+B+C}{1-R^{2}}$$

where R is the obscuration ratio, defined as $R=d_{obs}/D$; D is the diameter of the optical system, and $d_{obs}$ is the diameter of the obscured portion. Let:

(56) $$X=\frac{f_{x}}{f_{oc}},\;Y=\frac{X}{R},\;\alpha =\cos^{-1}\left(\frac{1+R^{2}-4X^{2}}{2R}\right)$$

where $f_{x}$ is the image spatial frequency, and $f_{oc}$ is the optical cutoff frequency of the lens, defined as $f_{oc}=1/\lambda F$, in which F represents the F-number of the system. The variables A, B, and C are respectively expressed by the following equations:

(57) $$A=\left\{\begin{array}{cc} \frac{2}{\pi }\left[\cos^{-1}(X)-X\sqrt{1-X^{2}}\right] & 0<X<1\\ 0 & \mathrm{otherwise} \end{array}\right.$$

(58) $$B=\left\{\begin{array}{cc} \frac{2R^{2}}{\pi }\left[\cos^{-1}(Y)-Y\sqrt{1-Y^{2}}\right] & 0<Y<1\\ 0 & \mathrm{otherwise} \end{array}\right.$$

(59) $$C=\left\{\begin{array}{cc} -2R^{2} & 0<X\leq (1-R)/2\\ \frac{2R}{\pi }\sin\alpha +\frac{1+R^{2}}{\pi }\alpha -\frac{2(1-R^{2})}{\pi }\tan^{-1}[(\frac{1+R}{1-R})\tan(\frac{\alpha }{2})]-2R^{2} & (1-R)/2<X<(1+R)/2\\ 0 & X\geq (1+R)/2 \end{array}\right.$$

2. Diffraction-limited MTF without Obscuration

For an unobstructed circular aperture, the MTF is a circularly symmetric function:

(60) $$MTF_{diff}\left(f_{x}\right)=\left\{\begin{array}{l} \frac{2}{\pi }\left[\cos^{-1}\left(\frac{f_{x}}{f_{oc}}\right)-\frac{f_{x}}{f_{oc}}\sqrt{\left(1-{\left(\frac{f_{x}}{f_{oc}}\right)}^{2}\right)}\right],f_{x}\leq f_{oc}\\ 0,f_{x}>f_{oc} \end{array}\right.$$

3. Aberration MTF

For the aberration-related MTF, when $f_{x}\leq f_{oc}$:

(61) $$MTF_{aberration}(f_{x})=1-{\left(\frac{W_{rms}}{A}\right)}^{2}[1-4{\left(\frac{f_{x}}{f_{oc}}-\frac{1}{2}\right)}^{2}]$$

where $W_{rms}$ denotes the root-mean-square (RMS) wavefront error. The relationship between the RMS wavefront error and the peak-to-valley (P-V) wavefront error ($W_{pp}$) is given by $W_{rms}=W_{pp}/3.5$, with the constant A=0.18. This empirical MTF formula is applicable to cases with small wavefront errors, specifically where $W_{pp}<0.5$.

4. Defocus MTF

The modulation MTF caused by defocus is expressed as:

(62) $${\mathrm{MTF}}_{defocus}=\frac{2J_{1}\left(\pi \kappa f_{N}/F\right)}{\pi \kappa f_{N}/F}$$

where $J_{1}$ is the first-order Bessel function; F is the F-number of the optical system; k is the defocus amount (unit: mm); $f_{N}$ is the Nyquist frequency. After precise focusing, the defocus MTF can be considered as 1.

5. Other MTF Factors

Assembly and fabrication also cause MTF degradation, which follows a Gaussian function:

(63) $$MTF_{\mathrm{Assembly}}=\exp\left[4\mathrm{lg}\left(0.5a\right){\left(\frac{f_{x}}{f_{oc}}\right)}^{2}\right]$$

(64) $$MTF_{\mathrm{fabrication}}=\exp\left[4\mathrm{lg}\left(0.5b\right){\left(\frac{f_{x}}{f_{oc}}\right)}^{2}\right]$$

where a and b are the assembly and fabrication factors, respectively. The spatial effect caused by the optical system can be obtained by multiplying the various MTF factors:

(65) $$MTF_{opt}=\cdot MTF_{diff}\cdot MTF_{aberration}\cdot MTF_{defocus}\cdot MTF_{\mathrm{Assembly}}\cdot MTF_{\mathrm{fabrication}}$$

#### 3.3.2. Detector MTF

The effects influencing the detector MTF primarily include the spatial integration of pixel size and the photoelectron diffusion effect.

(66) $$MTF_{cmos}=MTF_{P}\cdot MTF_{DIFFUSION}$$

6. Space integral

During the detector imaging process, the image function is continuously intercepted by an aperture function. This local averaging process can be modeled as a convolution of the input signal with the sampling aperture function. The sampling aperture is typically regarded as a rectangular function, as detailed below:

(67) $$S(x,y)=G\cdot N\cdot T\cdot A_{D}\int_{\lambda_{\min}}^{\lambda_{\max}}\frac{\pi \tau_{o}(\lambda )}{4F^{2}}(1-\varepsilon )\cdot L(x,y,\lambda )\cdot \mathrm{QE}\left(\lambda \right)\cdot pd\lambda +\frac{bias}{CCE}$$

The integral is solved using the rectangular rule as follows:

(68) $$S(x,y)=G\cdot L(x,y)\frac{\pi \tau_{o}}{4F^{2}}(1-\varepsilon )\cdot A_{D}\cdot T\cdot N\cdot \mathrm{QE}\frac{c}{h\lambda_{m}}+\frac{bias}{CCE}$$

Taking stray light and irradiance non-uniformity into account, the corrected signal model is expressed as follows:

(69) $$Se(x,y)=G\left[L(x,y)\frac{\pi \tau_{o}}{4F^{2}}(1-\varepsilon )\cdot t\cdot \cos^{4}\omega +E_{\mathrm{internal}}\left(x,y\right)\right]A_{D}\cdot T\cdot N\cdot \mathrm{QE}\frac{\lambda_{m}}{hc}+\frac{bias}{CCE}$$

where L(x,y) is the radiance; $\tau_{o}$ is the average transmittance; F is the F-number; ε is the area obscuration factor; t is the vignetting coefficient; ω is the field of view (FOV) angle; $E_{\mathrm{internal}}$ is the image plane irradiance of stray light (related to the stray light coefficient VGI, where $E_{\mathrm{internal}}=L*VGl$); $A_{D}$ is the detector pixel area; T is the integration time; QE is the average quantum efficiency; N is the number of integration stages (or TDI stages); c is the speed of light; h is Planck’s constant; $\lambda_{m}$ is the center wavelength; bias is the offset; CCE is the photoelectric conversion efficiency.

The expression for the vignetting coefficient *t* is given as follows:

(70) $$t=\left\{\begin{array}{cc} 1 & h\leq r_{2}-r_{1}\\ \begin{array}{l} 1-\frac{{r_{1}}^{2}+h^{2}-{r_{2}}^{2}}{2\pi h{r_{1}}^{2}}\sqrt{{r_{1}}^{2}-{\left(\frac{{r_{1}}^{2}+h^{2}-{r_{2}}^{2}}{2h}\right)}^{2}}+\frac{{r_{1}}^{2}-h^{2}-{r_{2}}^{2}}{2\pi h{r_{1}}^{2}}\sqrt{{r_{2}}^{2}-{\left(\frac{{r_{1}}^{2}+h^{2}-{r_{2}}^{2}}{2h}\right)}^{2}}\\ -\frac{1}{\pi }\arcsin\left(\frac{{r_{1}}^{2}+h^{2}-{r_{2}}^{2}}{2\pi h{r_{1}}^{2}}\right)+\frac{{r_{2}}^{2}}{\pi {r_{1}}^{2}}\arcsin\left(\frac{{r_{1}}^{2}-h^{2}-{r_{2}}^{2}}{2\pi h{r_{2}}^{2}}\right) \end{array} & r_{2}-r_{1}<h<d\tan\omega \\ 0 & h\geq d\tan\omega \end{array}\right.$$

where $r_{1}$ and $r_{2}$ are the radii of the projected circles formed by the oblique beam passing through the entrance window and the entrance pupil, respectively, and h is the distance between the two circle centers. To account for the non-linear response, the signal model is further refined as follows:

(71) $$Se\left(x,y\right)=\left\{\begin{array}{cc} a_{1}L & L\leq L_{1}\\ a_{2}(L-L_{1})+a_{1}L_{1} & L_{1}<L<L_{2}\\ 2^{-\left(a_{3}/L\right)} & L_{2}\leq L\leq L_{3} \end{array}\right.$$

where the selection of the threshold values $L_{1}$, $L_{2}$, and $L_{3}$ depends on the type of detector material, the operating spectral band, and the manufacturing process level.

7. Simulation Methodology

This simulation model is based on stellar magnitude. It requires converting the apparent magnitude of stars into physical luminous flux, and subsequently calculating the signal electron count through imaging mechanism modeling.

First, the stellar brightness—representing the grayscale distribution of the star on the image plane—is derived based on the apparent magnitude formula (the specific transformation relationship between apparent magnitude and image grayscale is detailed in the subsequent section on stellar characteristics). The energy distribution on the image plane is then simulated by incorporating the optical aperture, transmittance, and Point Spread Function (PSF) provided by the camera manufacturer.

Secondly, parameters such as the quantum efficiency, pixel size, and exposure time of the detector are utilized to convert the number of photons reaching the detector into the corresponding number of electrons.

Finally, by processing the data with circuit gain, dynamic range, and noise characteristic models, the signal intensity for each pixel is output. This completes the simulation transformation from stellar apparent magnitude to a two-dimensional image.

## 4. Experiment

The simulation algorithms were implemented in Python 3.11, leveraging its open-source scientific computing libraries. All experiments were conducted on a workstation equipped with a 13th Gen Intel Core i7-13650HX processor (2.60 GHz), 16 GB of RAM, and an NVIDIA GeForce RTX 4060 GPU. Given the high computational complexity associated with processing the massive Gaia catalog, GPU acceleration (parallel computing) was specifically employed to handle the coordinate transformations of stars from the celestial sphere to the image plane. Additionally, a block-wise computation strategy was implemented to partition the data. This effectively prevents GPU out-of-memory (OOM) errors when calculating the image plane positions for exceptionally dense regions, such as the Galactic Center. This combination of hardware-accelerated parallelization and memory management significantly optimizes the runtime, enabling the efficient computation of stellar coordinates in highly dense star fields.

### 4.1. Fidelity Testing

Based on the theoretical foundation and modeling established above, dense stellar simulation experiments were conducted to test the imaging of stars in deep-space scenarios. To verify the accuracy and effectiveness of the simulation results, the measured data and corresponding optical axis pointing from the GTC camera were utilized, information related to the GTC camera is shown in Table 1 [21,22]. The imaging results were simulated under identical imaging conditions and simulation models—using stars up to magnitude 15 from the star catalog based on the detection capability of the measured data—and then compared with the actual captured images. 

**Figure 7:**
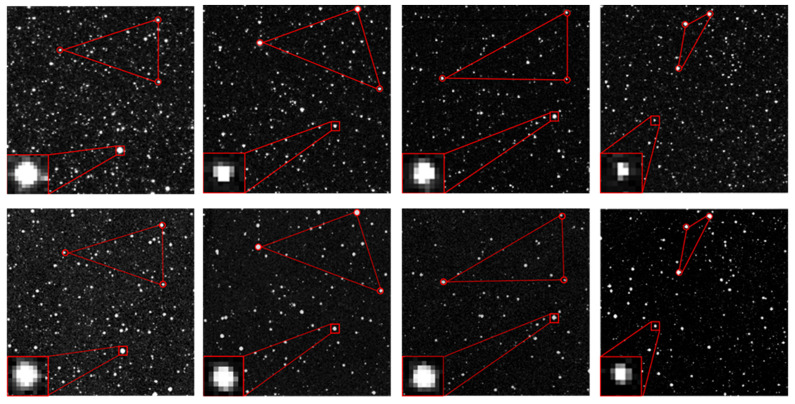
Comparison between experimental data and simulation results (**Top row**: real images captured by the GTC; **Bottom row**: simulated images of the corresponding field-of-view centers).

**Figure 8:**
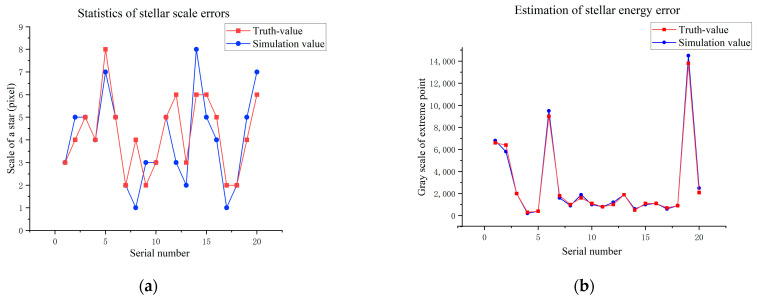
Statistical results of simulation errors for stellar scale and energy. (**a**) Statistics of stellar scale errors; (**b**) Estimation of stellar energy error.

**Figure 9:**
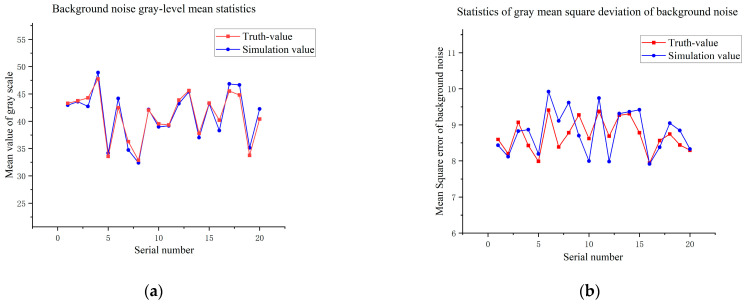
Statistical results of simulation errors for background noise. (**a**) Background noise gray-level mean statistics; (**b**) Statistics of gray mean square deviation of background noise.

As shown in Figure 7, both the visual effect of individual stellar targets and the relative positions between stars in the simulated image are highly consistent with the GTC measured image. To further validate the model’s fidelity through a statistically unbiased evaluation, we implemented a stratified random sampling strategy for both stellar targets and background regions. For stellar reference selection, detected stars were categorized into several magnitude bins to ensure the evaluation covers the full dynamic range of the sensor. Within each bin, 100 stars were randomly sampled across different quadrants to account for potential field-of-view non-uniformity, with each star cross-referenced to its simulated counterpart via Gaia catalog coordinates. Simultaneously, 20 × 20 pixel background regions were localized using a Monte Carlo-based random sampling approach, while strictly avoiding high-brightness stars and target signals to prevent energy leakage from biasing the noise statistics. The mean grayscale values and standard deviations within these regions were then analyzed, and the results are illustrated in Figure 8 and Figure 9:

The fidelity of the simulation is quantitatively validated through four critical dimensions. To ensure statistical robustness, the reported metrics represent average values derived from multiple independent simulation runs. These runs encompass various sky regions—strategically selected based on different stellar densities—and diverse detection conditions (which are further detailed in Section 4.2.4). First, the average SNR error (<10%) confirms the accuracy of our radiative transfer model. Second, the centroid precision (<0.01 pixels), achieved via bicubic interpolation, ensures sub-pixel geometric consistency. Third, the background statistical errors (<5% and <10%) prove that the noise floor and stochastic fluctuations mimic real sensor behavior. Finally, the energy concentration (>90%) validates the high-fidelity reconstruction of the PSF. Collectively, these metrics demonstrate that the simulated images are not merely visually similar, but physically equivalent to measured data for algorithm performance evaluation.

### 4.2. Simulation Experiments for Special Scenarios

#### 4.2.1. Simulation of Dim and Weak Targets

For the simulation of dim and weak targets, a directional Gaussian filter is employed to simulate the smearing (trailing) effect of moving targets within the image. The brightness of the target region is then enhanced based on the Signal-to-Noise Ratio (SNR) and local statistical characteristics. Finally, an integrated image containing the moving targets is synthesized. Let $v_{x}$ and $v_{y}$ be the velocity components. The formulas for calculating the smearing length and the smearing angle are as follows:

(72) $$\mathrm{tail}\text{\_}\mathrm{length}=0.2\times \sqrt{v_{x}^{2}+v_{y}^{2}}$$

(73) $$\theta =-\arctan\left(\frac{v_{y}}{v_{x}}\right)$$

**Figure 10:**
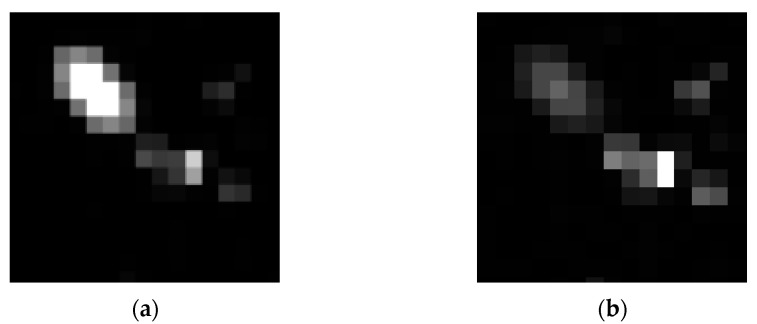
Simulation effects of targets with different signal-to-noise ratios. (**a**) target SNR = 9; (**b**) target SNR = 3.

Let $\sigma_{tar}$ be the standard deviation of the target region, $\mu_{avg}$ be the average background grayscale, and $I_{h>0}$ be the binary mask. The target brightness enhancement formula is given as follows and the simulation results are shown in Figure 10:

(74) $$D(x,y)=h(x,y)\cdot \mathrm{SNR}\cdot \sigma_{tar}\;+\mu_{avg}\;\cdot I_{h>0}\;(x,y)$$

#### 4.2.2. Platform Motion Simulation

**Figure 11:**
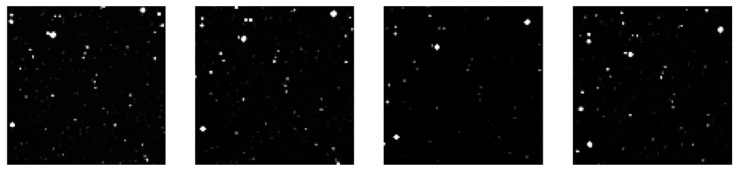
Demonstration of image motion effects (The time interval between successive frames is 8 s).

The impact of platform motion is primarily manifested in the image motion (smearing) of stars and targets. During simulation, it is necessary to evaluate the magnitude of background stellar movement, considering both stellar position displacement and image motion blur. Consequently, the coordinates of the stars and targets on the image plane must be updated frame by frame. The position update formulas are as follows:

(75) $$\begin{array}{l} x_{n}=\cos(\theta )\cdot L\cdot (n-1)+x_{1}\\ y_{n}=\cos(\theta )\cdot L\cdot (n-1)+y_{1} \end{array}$$

where $\left(x_{n},y_{n}\right)$ represents the image plane position of the star or target at the n frame, θ is the direction of image motion, and L is the image motion distance per frame. The image motion effect is illustrated in the Figure 11.

#### 4.2.3. Star–Target Overlap (Sticking) Simulation

**Figure 12:**
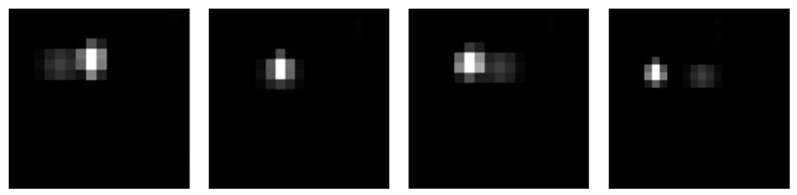
Target–star overlap during transit.

**Figure 13:**
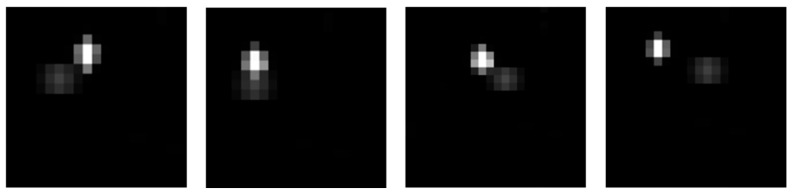
Target–star grazing during transit.

When the stellar density is high, overlapping (sticking) between the target and background stars is inevitable, necessitating the use of mask processing. The masking strategy is as follows: compare the grayscale values of the star and the target within the overlapping region; if the target’s energy in the overlap is higher than that of the star, the original region is replaced by the target’s pixels; otherwise, the original stellar pixels are retained. The logic for updating the image region is expressed as follows and the simulation results are shown in Figure 12 and Figure 13:

(76) $$\mathrm{Output}(x,y)=\left\{\begin{array}{cc} D(x,y) & \mathrm{if}\;D(x,y)>\mathrm{temp}\text{\_}\mathrm{img}(x,y)\;\\ \mathrm{temp}\text{\_}\mathrm{img}(x,y) & \mathrm{otherwise} \end{array}\right.$$

#### 4.2.4. Simulation of Different Detection Limits

**Figure 14:**
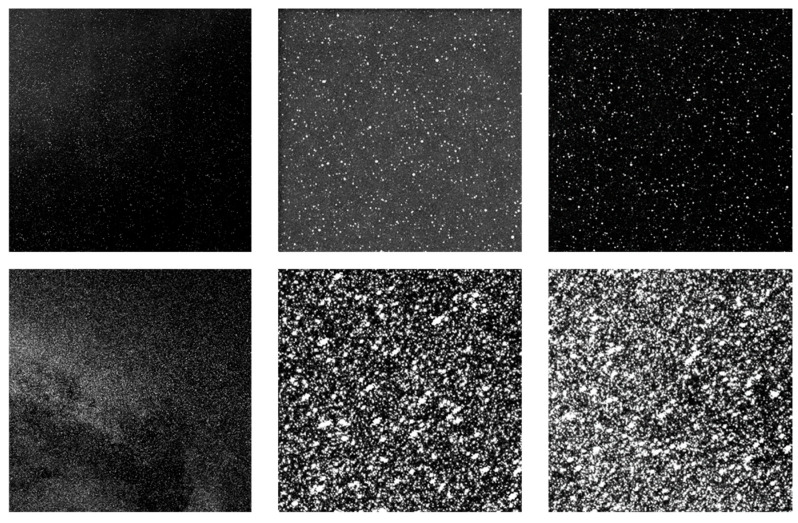
Comparative simulation results of different detection limits (**Top row**: simulation images and enlarged sub-regions for the 15th-magnitude detection limit; **Bottom row**: simulation images and enlarged sub-regions for the 22nd-magnitude detection limit).

Corresponding simulations were conducted to compare the performance under various detection limits of the detector. We performed comparative simulations for two scenarios: magnitude 15 at SNR 3 and magnitude 22 at SNR 3. It can be observed that under the 15-magnitude @ SNR 3 condition, the image spatial duty cycle is lower, and the overall image brightness is relatively dim. In contrast, under the 22-magnitude @ SNR 3 condition, the image spatial duty cycle is significantly higher, resulting in a higher overall brightness; furthermore, multiple stars may overlap within a single pixel, the simulation results are shown in Figure 14.

## 5. Conclusions

To address the critical requirement for high-fidelity data in high-orbit Space Situational Awareness (SSA) and deep-space target recognition, this paper presents a comprehensive simulation methodology for dense stellar backgrounds. By integrating the Gaia catalog with target Two-Line Element (TLE) data, we developed an end-to-end optoelectronic imaging model capable of accurately simulating faint signals, such as space debris and asteroids, against complex deep-space environments. Quantitative evaluations demonstrate that the proposed method achieves high fidelity across multiple metrics, including Signal-to-Noise Ratio (SNR), geometric precision, and background statistical characteristics, with all relative errors maintained below 10%.

Recognizing the computational challenges posed by the vast volume of the Gaia dataset—particularly when simulating high limiting magnitudes or large image scales—a strategic data optimization framework was implemented. The Gaia catalog was spatially partitioned into manageable sub-sets, with data storage streamlined to retain only essential simulation attributes: Right Ascension (RA), Declination (Dec), and apparent magnitude. Furthermore, the catalog was categorized into discrete subsets based on limiting magnitudes (e.g., 10, 14, 18, and 22), facilitating on-demand data loading for varied mission requirements. This hierarchical optimization significantly enhances data retrieval efficiency and ensures the scalability of the framework for generating high-density star maps.

Simulation results highlight the significant impact of signal aliasing and background interference on dim target detection within high-orbit environments. By covering a broad spectrum of scenarios—ranging from magnitude 15 to 22 with varying target dynamics—this system provides a robust platform for the design optimization of future space-based detection payloads, algorithm training, and limit-capability assessments.

## Figures and Tables

**Table 1:** Description of GTC Camera Measured Data.

Camera Information	Parameter
Image Size	6144 × 6144
Field of View	21° × 21°
Detection Capability	14.8~15
Center Pointing	(299.91, 40.09); (291.32, −5.13); (176.62, −0.16)

## Data Availability

The raw data supporting the conclusions of this article will be made available by the authors on request.
